# Running Behind “POPO”—Impact of Predictors of Poor Outcome for Treatment Stratification in Pediatric Crohn's Disease

**DOI:** 10.3389/fmed.2021.644003

**Published:** 2021-08-27

**Authors:** Jan de Laffolie, Klaus-Peter Zimmer, Keywan Sohrabi, Almuthe Christina Hauer

**Affiliations:** ^1^Department of General Pediatrics and Neonatology, Pediatric Gastroenterology, University of Giessen, Giessen, Germany; ^2^Department Medical IT, Technical University Giessen, Giessen, Germany; ^3^Department of Pediatrics and Adolescent Medicine, Medical University Graz, Graz, Austria

**Keywords:** pediatric Crohn's disease, outcome parameters, predictors of a poor prognosis, patient registry, big data

## Abstract

**Background and Aims:** Intensifying therapy for Paediatric Crohn's Disease (CD) by early use of immunomodulators and biologics has been proposed for cases in which predictors of poor outcome (POPO) were present. We investigated therapy stratifying potential comparing POPO-positive and -negative CD patients from CEDATA-GPGE®, a German-Austrian Registry for Paediatric Inflammatory Bowel disease.

**Methods:** CD patients (1–18 years) registered in CEDATA-GPGE® (2004–2018) within 3 months of diagnosis and at least two follow-up visits were included. Disease course and treatments over time were analysed regarding positivity of POPO criteria and test statistical properties.

**Results:** 709/1084 patients included had at least one POPO criterion (65.4%): 177 patients (16.3%) had persistent disease (POPO2), 581 (53.6%) extensive disease (POPO3), 21 (1.9%) severe growth retardation POPO4, 47 (4.3%) stricturing/penetrating disease (POPO6) and 122 (11.3%) perianal disease (POPO7). Patients with persistent disease differed significantly in lack of sustained remission >1 year (Odd Ratio (OR) 1.49 [1.07–2.07], *p* = 0.02), patients with initial growth failure in growth failure at end of observation (OR 51.16 [19.89–131.62], *p* < 0.0001), patients with stricturing and penetrating disease as well as perianal disease in need for surgery (OR 17.76 [9.39–33.58], *p* < 0.001; OR 2.56 [1.58–4.15], *p* < 0.001, respectively). Positive Predictive Value for lack of sustained remission was >60% for patients with initial growth failure, persistent or stricturing/penetrating disease.

**Conclusion:** Predictors of poor outcome with complicated courses of disease were common in CEDATA-GPGE®. An early intensified approach for paediatric CD patients with POPO-positivity (POPO2-4, 6-7) should be considered, because they have an increased risk to fare poorly.

## Introduction

Medical management in paediatric Crohn's Disease (CD) still consists primarily of a step-up approach, with immunomodulators or biologics often being used only after other options have failed. Although these medications may induce and maintain remission with catch-up growth (1), this frequently failed to compensate for already acquired growth retardation (1–4). Optimization of therapeutic strategies for and identification of high-risk patients who require intensified therapy earlier is therefore mandatory.

The consensus guidelines of ESPGHAN/ECCO therefore describe predictors of poor outcome (POPO) as means for risk stratification (2). These are deep colonic ulcerations on endoscopy (POPO1), persistent severe disease despite adequate induction therapy (POPO2), extensive disease (POPO3), marked growth retardation [<-2.5 Height Standard Deviation Score (SDS); POPO4], severe osteoporosis (POPO5), stricturing and penetrating disease at onset (POPO6) and also perianal disease (POPO7). Whether these predictors may be used for definite risk stratification in paediatric CD, however, has not been substantiated to date by large scale data.

In 2004, CEDATA-GPGE® was founded as Registry of the Society for Paediatric Gastroenterology and Nutrition of German speaking countries (GPGE), enrolling paediatric Inflammatory Bowel Disease (IBD) patients in Germany and Austria. The main objective of this observational study was to evaluate the POPO potential for risk stratification by characterising and comparing disease course and treatment of POPO-positive vs. -negative patients from CEDATA-GPGE® and to describe “POPO” criteria test statistical properties.

## Patients and Methods

All CD patients (eligible 1–18 years of age, diagnosis in one of the participating centres) registered between 2004 and 2018 in CEDATA-GPGE® were included if documented in the registry within 3 months of diagnosis (to avoid recall bias) and at least two follow up visits, independent of therapeutic strategy chosen. Patients with delayed diagnostic workup (initial workup not completed within 3 months of diagnosis in the registry) were excluded. CEDATA-GPGE® is a prospective, multicentre registry for paediatric IBD in German speaking countries approved by ethic committees of all participating centres (5). Data entry is encouraged at least every 6 months by means of a secure and easy-to-use online registry environment (eSupplementary Example Figures). Diagnosis was based on Porto criteria (6) and while deep colonic ulcerations (POPO1) and osteoporosis (POPO5) had not been recorded systematically, the remaining 5 of 7 POPO criteria were evaluated in all patients, POPO3 extensive disease was approximated by L3+-L4a/b (Table 1). POPOs were considered positive when present at diagnosis, outcome measures were considered outcomes when they appeared at any point in time after diagnosis (for survival analysis, the first 4 weeks were eliminated from outcomes). For purposes of cohort homogeneity, patients with disease onset <1 year of age were excluded.

**Table 1:** “POPO” groups and patients' characteristics.

**POPO**	**Description**	**Definition in Registry**
“POPO”-2	Persistent disease	- on physician general assessment ^†^ - at 12 weeks after diagnosis ^‡^ - under recommended treatments ^§^
“POPO”-3	Extensive disease	- L3 Paris classification with/without upper gastrointestinal tract involvement
“POPO”-4	Severe growth retardation	- body height < -2.5 SDS ^¶^
“POPO”-6	Stricturing/penetrating disease	- radiologic/endoscopic assessment
“POPO”-7	Perianal disease	- excluding simple tags and fissures, clinical assessment (7)

† *Physician general assessment: By 4-item assessment scale (remission – mild – moderate – severe disease activity, levels 3–4) used in other large registries (8)*.

‡ *12 weeks after inclusion: Time point for relevant information on early disease course*.

§ *Recommended treatments: Exclusive enteral nutrition, steroids, 5-ASA, methotrexate, azathioprine/6-mercaptopurin, infliximab*.

¶ *Body height < -2.5 SDS, according to reference values from the “KIGGs Study,” a representative nationwide health survey (9)*.

A sensitivity analysis for the exclusion of younger patients in our main findings was conducted to evaluate if exclusion of patients with <1 or 2 years of age would change results. Enrolled patients were divided into two groups: Patients fulfilling at least one criterion were **POPO-positive**, and those without fulfilling any criterion **POPO-negative**. Both groups were compared in the context of basic characteristics such as age and gender, disease activity (Paediatric Crohn's Disease Activity Index PCDAI) (6) assessment of diagnostic latency (time from onset of symptoms to diagnosis as described by the patients or parents), disease presentation and course: Growth failure (Height < −2.5 SDS, Table 1) and failure to gain weight (history of failure to gain weight, failure to thrive) were documented as presenting symptoms, severe growth retardation was one of the predictors to be investigated and growth failure was again used as an endpoint toward which predictive measurements were evaluated. Therapy over a maximum of 5 years was analysed additionally, with restriction criteria to azathioprine as an immunomodulatory and infliximab as a biologic agent. Since accelerated step-up treatment strategy has to be decided earlier than at 1 year, we defined lack of response to adequate induction therapy at 3 months as “persistent disease.” Adequate induction therapy was defined as documented acceptable therapies for induction of paediatric CD like exclusive enteral nutrition, systemic steroids or infliximab/Adalimumab. Patients between 1 and 6 years received subgroup analysis (very early onset IBD).

For each POPO criterion sensitivity, specificity, positive and negative predictive values [PPV and NPV, respectively (resp.)] including 95% confidence intervals for odds ratios were calculated with regard to the variety of outcome parameters. These were extraintestinal manifestations (EIM), as recorded in the online registry (uveitis, arthritis, skin manifestations, hepatobiliary involvement, others), lack of sustained remission for >1 year (sustained remission represented by at least two physician global assessments indicating full remission at least 365 days apart without intermittent inflammatory activity or intensified therapy), presence of abscess, fistula or stenosis (including perianal fistula), surgery (both as recorded in the registry by the treating physician) and growth failure at the end of observation (body height < −2.5 SDS). We also analysed differences of negative outcomes comparing early vs. late (< = 3 vs. >3 months from diagnosis, dichotomized for analysis) start of azathioprine or infliximab. This cut off was chosen according to relevant literature in the field.

To address longterm outcome survival analysis for all predictor positive groups vs. negative groups was performed (Kaplan Meyer Survival Curve, Log Rank Analysis, Cox Regression Analysis where follow-up time differed). Events within the first four weeks after diagnosis were eliminated from Kaplan Meyer and Log Rank Analysis.

Statistical analysis was performed on the SAS Version 9.4 (SAS-Institute, North Carolina) and R (CRAN, Vers 3.6.3). Fisher exact-test, chi-square-and Wilcoxon tests were used for comparison variables between groups. The *p*-value for statistical significance was defined as <0.05.

## Results

### Baseline Characteristics

**Figure 1:**
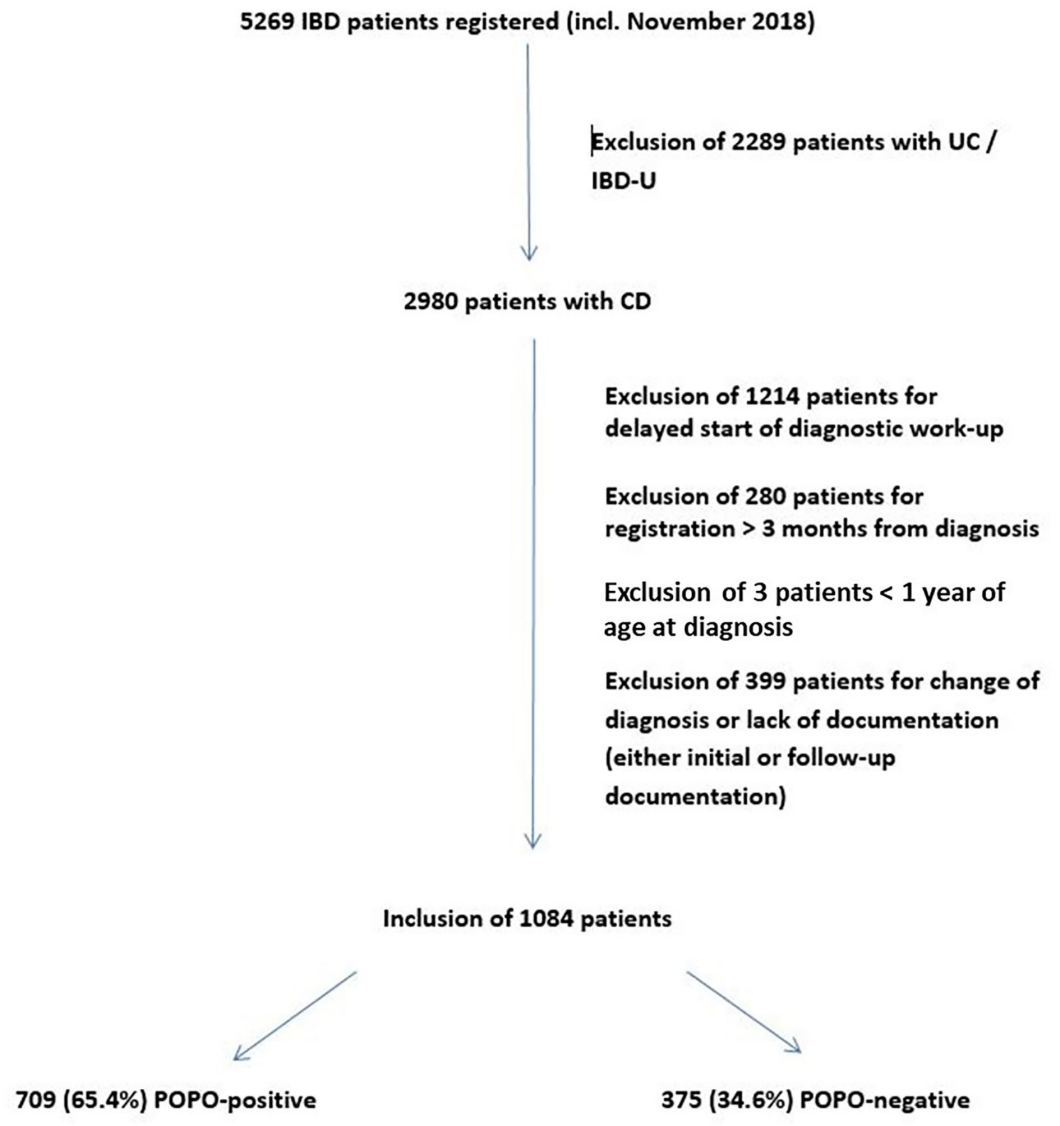
Study population.

Of 5,269 IBD patients (age 0–18 years) registered in “CEDATA-GPGE®” from 2004 to 2018, 2,980 patients were diagnosed with CD and 1,084 were included in the study (Figure 1). 50 (4.6%) of them were between 1 and 6 years of age. The sensitivity analysis on the lower age margin showed no difference between the exclusion at 1 or 2 years of age.

**Figure 2:**
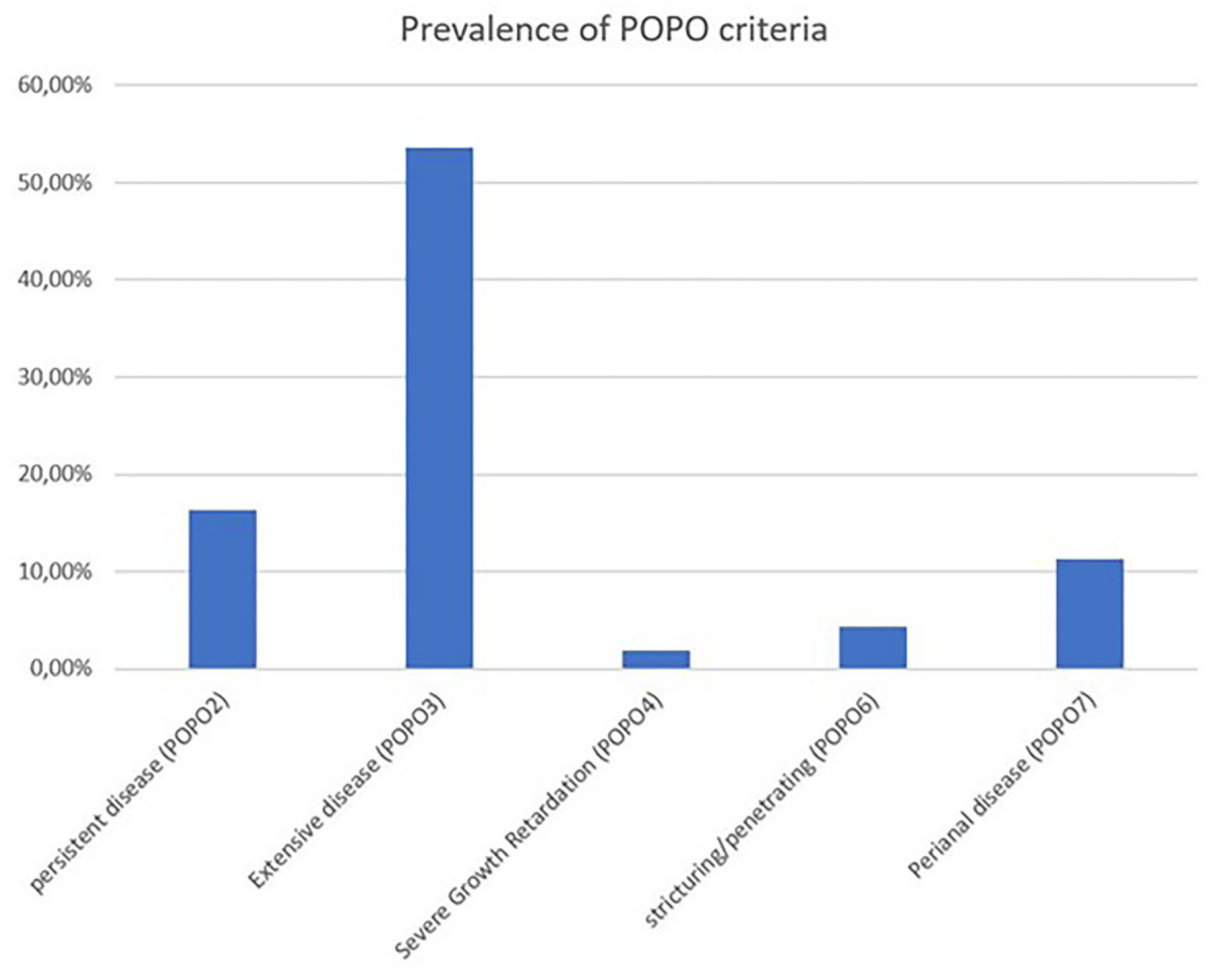
Prevalence of POPO criteria. Prevalence of POPO criteria at initial diagnosis of paediatric CD patients from CEDATA GPGE registry.

The median follow-up was 10 visits [interquartile range (IQR) 5–15 visits, max 94 visits] over a time period of a median of 769 days (IQR 275–1,595 days, max 3,570 days). In 709 patients (65.4%; 70% between 1 and 6 years) at least one POPO-criterion was found. 177 (16.3%; 45.7% between 1–6 years) patients had persistent severe disease despite recommended induction therapy (POPO2). 581 patients (53.6%; 68.6% between 1 and 6 years) showed extensive disease (POPO3), 21 (1.9%) had severe growth retardation (POPO4) and 47 (4.3%) stricturing or penetrating disease (POPO6). Perianal disease (POPO7) was recorded in 122 patients (11.3%; Figure 2). While patients between 1 and 6 years of age were significantly more often POPO2- and−3-positive than the older age group, only few of them were POPO4-, POPO-6, and POPO-7-positive, resp.

### POPO-Positive vs. POPO- Negative Groups

#### Characteristics

POPO-positive and -negative patients did not differ significantly in age and gender, and just reached significance regarding PCDAI at diagnosis (23.3 vs. 19.3, *p* = 0.05) and diagnostic latency (8.9 vs. 7.9 months; *p* < 0.05; Table 2, missing values did not differ significantly between groups).

**Table 2:** Characteristics of POPO^*^-positive and POPO-negative patient groups.

**Characteristics**	**POPO positive (*n* = 709)**	**POPO negative (*n* = 375)**	***P***
Male	419 (59.1%)	217 (57.9%)	0.70
Mean age at diagnosis (years ± standard deviation SD)	12.4 ± 3.2	12.9 ± 3.1	0.06
Mean diagnostic latency (months ± SD)	8.9 ± 12.6 (*n =* 687)	7.9 ± 12 (*n =* 361)	**0.05**
PCDAI^*^, ^**^ at diagnosis mean ± SD	23.3 ± 13.1 (*n =* 313)	19.3 ± 13.6 (*n =* 171)	**0.05**
Azathioprine	528 (74.5%)	260 (69.3%)	0.07
Infliximab	154 (21.7%)	76 (20.3%)	0.6
mean duration of follow-up (months ± SD)	36.00 ± 28.4	34.05 ± 28.0	0.28

* *POPO, predictors of poor outcome*;

** *PCDAI, paediatric crohn's disease acitivity index; SD, standard deviation. The bold values indicates p < 0.05*.

#### Symptoms at Diagnosis

On diagnosis, POPO-positive patients had significantly reduced appetite and limitation in activities in comparison to POPO-negative patients (*p* = 0.04; *p* = 0.03, resp., Supplementary Table e3), but did not differ significantly regarding all other presenting symptoms evaluated (abdominal pain, diarrhoea, blood in stool, failure to gain weight, growth failure, fever, anaemia, loss of appetite, EIM; Supplementary Table e3).

#### Analysis per POPO-Group: POPO2-Positive

**Figure 3:**
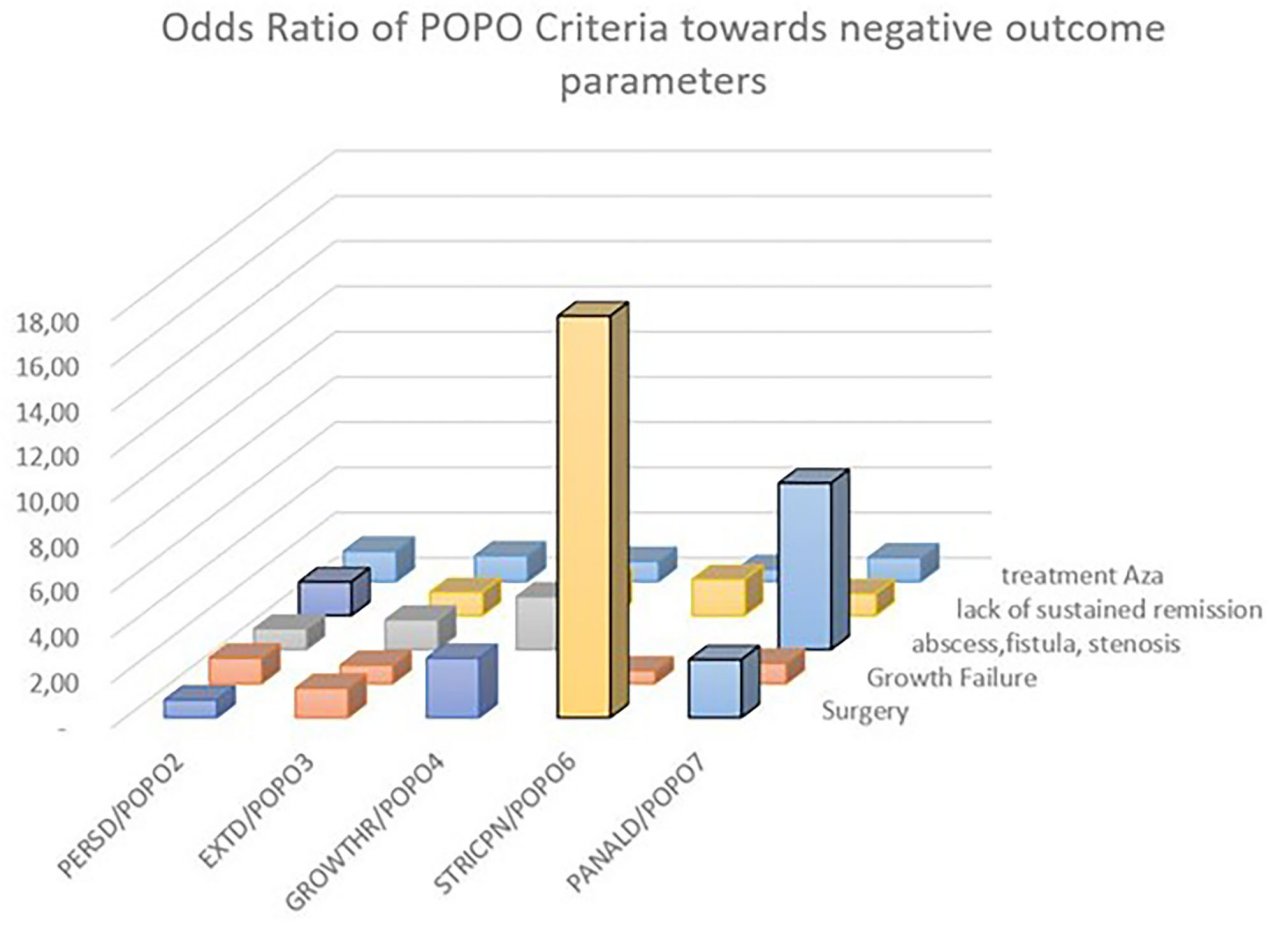
Odds ratio of POPO toward negative outcome parameters. Statistically significant OR 95% CI are marked by bold contours; OR for growth retardation/persistent growth failure 51.16 [19.89–131.62] and stricturing/penetrating behaviour/abscess fistula stenosis 276.31 [37.82–2018.97] were removed for better overview.

(persistent disease) patients had a significantly higher risk for lack of sustained remission >1 year (OR 1.49 [1.07–2.07], *p* = 0.02), with a PPV of 61.02% (Table 3; Figure 3). A majority of patients with initial severe growth retardation (**POPO4 positive**) continued to have growth failure, differing significantly in comparison to POPO-negative patients (OR 51.16 [19.89–131.62], *p* < 0.0001). The PPV for lack of sustained remission was 61.9% (Table 3). Patients with stricturing or penetrating disease behaviour (**POPO6-positive**) had significantly less treatment with azathioprine than POPO negative patients (0.54 [0.3–0.98]; *p* < 0.04) and EIM (OR 0.33 [0.17–0.66], *p* = 0.001). They developed abscess, fistula, or stenosis significantly more often (OR 276.31 [37.82–2018.97], *p* < 0.0001) and also had surgery significantly more often (OR 17.76 [9.39–33.58], *p* < 0.001; Table 3; Supplementary Table e4; Figure 3). PPV for lack of sustained remission was 63.83% (Table 3). **POPO7-positive** patients (perianal disease) had significantly increased risks for abscess, fistula, or stenosis (OR 7.36 [4.93–11.0], *p* < 0.0001) and surgery (OR 2.56 [1.58–4.15], *p* < 0.001; Table 3; Supplementary Table e4; Figure 3). Specificity and negative predictive value toward negative outcomes were high in case of POPO2, POPO4, POPO6 and POPO7 (Supplementary Table e2).

**Table 3:** POPO-positivity and predictive properties.

**POPO2 Follow-up Avg. 32.67 +- 26.6 months Endpoint**	**Sensitivity**	**Specificity**	**PPV**	**NPV**	**Odds Ratio [95%CI]**	***p*-value compared to POPO neg**.
Surgery	16/118 (13.56%)	805/966 (83.33%)	16/177 (9.04%)	805/907 (88.97%)	0.78 [0.45–1.36]	0.39
Growth failure	7/39 (17.95%)	875/1,045 (83.73%)	7/177 (3.95%)	875/907 (96.47%)	1.13 [0.49–2.59]	0.78
Abscess, fistula, stenosis	30/194 (15.46%)	743/890 (83.48%)	30/177 (16.95%)	743/907 (81.92%)	0.92 [0.6–1.42]	0.72
Lack of sustained remission	108/573 (18.85%)	442/511 (86.5%)	108/177 (61.02%)	442/907 (48.73%)	1.49 [1.07–2.07]	**0.02**
Escalate to Azathioprine	137/788 (17.39%)	256/296 (86.49%)	137/177 (77.40%)	256/907 (28.22%)	1.35 [0.92–1.97]	0.12
Escalate to Infliximab	40/230 (17.39%)	717/854 (83.96%)	40/177 (22.60%)	717/907 (79.05%)	1.10 [0.75–1.62]	0.62
EIM	92/507 (18.15%)	492/577 (85.27%)	92/177 (51.98%)	492/907 (54.24%)	1.28 [0.93–1.77]	0.13
**POPO3 Follow-up Avg. 36.65** **+- 28.6 months Endpoint**	**Sensitivity**	**Specificity**	**PPV**	**NPV**	**Odds Ratio [95%CI]**	***p*****-value compared to POPO neg**.
Surgery	70/118 (59.32%)	455/966 (47.10%)	70/581 (12.05%)	455/503 (90.46%)	1.3 [0.88-1.91]	0.19
Growth failure	19/39 (48.72%)	483/1,045 (46.22%)	19/581 (3.27%)	483/503 (96.02%)	0.82 [0.43–1.55]	0.53
Abscess, fistula, stenosis	115/194 (59.28%)	424/890 (47.64 %)	115/581 (19.79%)	424/503 (84.29%)	1.32 [0.96–1.82]	0.08
Lack of sustained remission	310/573 (54.1%)	240/511 (46.97%)	310/581 (53.36%)	240/503 (47.71%)	1.04 [0.82–1.33]	0.72
Escalate to Azathioprine	430/788 (54.57%)	145/296 (48.99%)	430/581 (74.01%)	145/503 (28.83%)	1.15 [0.88–1.51]	0.30
Escalate to Infliximab	125/230 (54.35%)	398/854 (46.60%)	125/581 (21.51%)	398/503 (79.13%)	1.04 [0.76–1.39]	0.80
EIM	280/507 (55.23%)	276/577 (47.83%)	280/581 (48.19%)	276/503 (54.87%)	1.13 [0.89–1.44]	0.32
**POPO4 Follow-up Avg. 32.43** **+- 27.1 months Endpoint**	**Sensitivity**	**Specificity**	**PPV**	**NPV**	**Odds Ratio [95%CI]**	***p*****-value compared to POPO neg**.
Surgery	5/118 (4.24%)	950/966 (98.34%)	5/21 (23.81%)	950/1,063 (89.37%)	2.63 [0.94–7.31]	0.05
Growth failure	12/39 (30.77%)	1,036/1,045 (99.14%)	12/21 (57.14%)	1,036/1,063 (97.46%)	51.16 [19.89–131.62]	**<0.0001**
Abscess, fistula, stenosis	7/194 (3.61%)	876/890 (98.42%)	7/21 (33.33%)	876/1,063 (82.41%)	2.34 [0.93–5.88]	0.06
Lack of sustained remission	13/573 (2.27%)	503/511 (98.43%)	13/21 (61.9%)	503/1,063 (47.3%)	1.46 [0.6–3.55]	0.40
Escalate to Azathioprine	15/788 (1.90%)	290/296 (97.97%)	15/21 (71.43%)	290/1,063 (27.28%)	0.94 [0.36–2.44]	0.9
Escalate to Infliximab	6/230 (2.61%)	839/854 (11.71%)	6/21 (28.57%)	839/1,063 (78.93%)	1.50 [0.57–3.91]	0.41
EIM	14/507 (2.76%)	570/577 (98.7%)	14/21 (66.66%)	570/1,063 (53.62%)	2.31 [0.93–5.78]	0.07
**POPO6 Follow-up Avg. 21.8** **+- 21.9 months Endpoint**	**Sensitivity**	**Specificity**	**PPV**	**NPV**	**Odds Ratio [95%CI]**	***p*****-value compared to POPO neg**.
Surgery	16/118 (13.56%)	935/966 (96.79%)	16/47 (34.04%)	935/1,037 (90.16%)	17.76 [9.39–33.58]	**<0.001**
Growth failure	1/39 (2.56%)	999/1,045 (95.60%)	1/47 (2.13%)	999/1,037 (96.33%)	0.57 [0.08–4.25]	0.58
Abscess. fistula. stenosis	46/194 (23.71%)	889/890 (99.89%)	46/47 (97.87%)	889/1,037 (85.73%)	276.31 [37.82–2018.97]	**<0.0001**
Lack of sustained remission	30/573 (5.23%)	494/511 (96.67%)	30/47 (63.83%)	494/1,037 (47.64%)	1.61 [0.87–2.95]	0.12
Escalate to Azathioprine	28/788 (3.55 %)	277/296 (93.58%)	28/47 (59.57%)	277/1,037 (26.71%)	0.54 [0.3–0.98]	**0.04**
Escalate to Infliximab	13/230 (5.65%)	820/854 (96.02%)	13/47 (27.66%)	820/1,037 (79.07%)	1.44 [0.75–2.79]	0.27
EIM	11/507 (2.17%)	541/577 (93.76%)	11/47 (23.40%)	541/1,037 (9.64%)	0.33 [0.17–0.66]	**0.001**
**POPO7 Follow-up Avg. 36.7** **+- 29.0 months Endpoint**	**Sensitivity**	**Specificity**	**PPV**	**NPV**	**Odds Ratio [95%CI]**	***p*****-value compared to POPO neg**.
Surgery	26/118 (22.03%)	870/966 (90.06%)	26/122 (21.31%)	870/962 (90.44%)	2.56 [1.58–4.15]	**<0.001**
Growth failure	4/39 (10.26%)	927/1,045 (88.71%)	4/122 (3.28%)	927/962 (96.36%)	0.9 [0.32–2.57]	0.84
Abscess. fistula. stenosis	57/194 (29.38%)	833/890 (93.6%)	57/122 (46.72%)	833/962 (86.59 %)	7.36 [4.93–11.0]	**<0.0001**
Lack of sustained remission	64/573 (11.17%)	453/511 (88.65%)	64/122 (52.46%)	453/962 (47.09%)	0.98 [0.67–1.43]	0.93
Escalate to Azathioprine	90/788 (11.42%)	264/296 (89.19%)	90/122 (73.77%)	264/962 (27.44%)	1.06 [0.69–1.63]	0.78
Escalate to Infliximab	34/230 (14.78%)	766/854 (89.70%)	34/122 (27.87%)	766/962 (79.63%)	1.51 [0.99–2.31]	0.06 (0.056)
EIM	59/507 (11.64%)	514/577 (89.08%)	59/122 (48.36%)	514/962 (53.43%)	1.07 [0.74–1.57]	0.71

*The bold values indicates p < 0.05*.

#### Therapy

Analysis of therapy according to POPO-positivity revealed POPO-positive patients being treated significantly more often with infliximab (11.1 vs. 7.3%; *p* = 0.047, Supplementary Table e1) than POPO-negative patients in the first year from diagnosis. There were no significant differences regarding infliximab treatment in the second year, nor regarding azathioprine therapy in the first two years from diagnosis (64.4 vs. 61.5%; Supplementary Table e1) and beyond.

#### Survival Analysis

**Figure 4:**
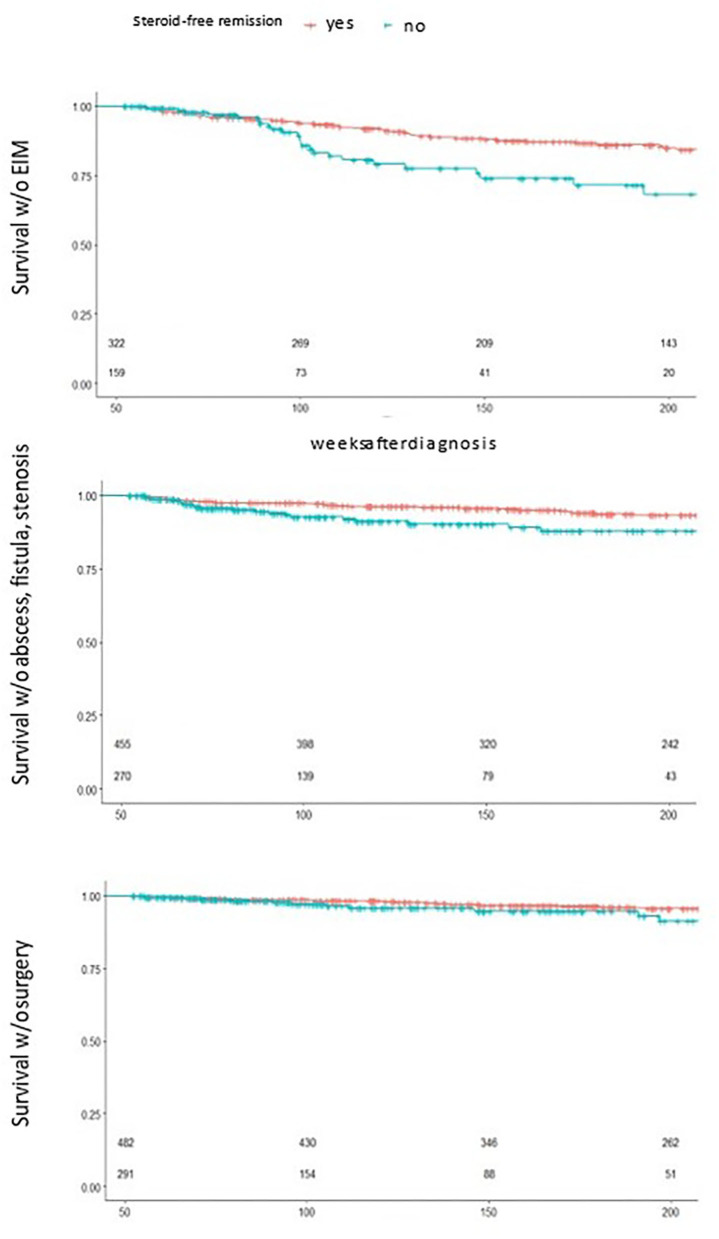
Survival analysis by 1 year steroid free remission. Statistically significant reduced event free survival for children who did not reach 1 year steroid free survival toward EIM(top), abscess, fistula and stenosis (middle) and surgery (bottom), small numbers n patients under risk.

In search for the most relevant predictor, the group of patients who did not ever reach steroid free remission over 1 year stood out (Figure 4). This group of children showed a significantly decreased event free survival over 200 weeks toward surgery, EIM and abscess, fistula and stenosis, a predictive value that was not reached by another one of the initially proposed criteria.

## Discussion

A modern approach to CD should be influenced by the patient's underlying prognosis and there are several outcomes aimed for with CD: Remission, in adults commonly graded by the Crohn's disease activity index (10) or Harvey Bradshaw index scoring system (8), is one such outcome. Other outcomes include the avoidance of surgery, endoscopic mucosal remission, and reduction of long-term bowel damage. Whether additional analysis of genotypes, antimicrobial serologies, ileal gene expression, and faecal microbiota might be used for predicting a complicated CD course in children has been addressed recently by Kugathasan et al. in a multicentre inception cohort study. Specific bacteria, i.e., *Ruminococcus* and *Veillonella*, were found to be implicated in stricturing and penetrating complications, resp., and the signature of upregulated ileal genes controlling extracellular matrix production associated with stricturing disease, thus supporting the usefulness of risk stratification at diagnosis (11).

We therefore aimed at analysing POPO longitudinally by using data from the CEDATA-GPGE® set, the second largest prospective registry of paediatric IBD worldwide, and, as is mostly true with registries, with this bringing in an additional value in its own, reflecting daily life clinical care (12). To ensure data quality, which might have been impaired due to the registry's long term nature and potential selection bias with only tertiary centres participating, we tried to minimise recall bias of initial presentation and missing of early events, in that only patients registered within 3 months of diagnosis were analysed.

In our study we found two thirds of all patients, and 70% of those younger than 6 years, were POPO-positive, in contrast to observations from a French population-based cohort study with complicated disease behaviour in only 31% of paediatric CD patients at diagnosis (13). We are thus confident that our cohort is suitable for testing the predictive properties of POPO criteria. POPO-positive patients were only slightly younger than POPO-negative patients, and the age difference of only a few months might not be clinically relevant (14, 15). The higher PCDAI at diagnosis could indicate a more severe disease course, as shown for at least the first year after diagnosis in a report from Hungary (16). However, we did not analyse initial PCDAI separately as a predictor for long term outcome, because, in accordance with published data, its preliminary analysis did not yield a highly significant effect (16).

More than 16% of our patients had persistent severe disease despite adequate induction therapy (POPO2-positive) while the rates for similar patient groups were reported as only 2.1% after 1 year (PCDAI >31) in a Hungarian registry (16) and 5% by Dubner et al. (17). These differences could be explained by other observational periods and disease activity scores. Since an accelerated step-uptreatment strategy has to be decided earlier than at 1 year, we defined lack of response to induction therapy at 3 months as “persistent disease.” This definition meets the demand for an early predictive parameter more adequately.

With almost 54% of patients suffering extensive disease (POPO3-positive) our findings are in keeping with own previous reports (3) and data from the EUROKIDS-Registry (17, 18), showing the majority of paediatric CD patients to have pancolonic involvement. However, POPO3-, as well as POPO2-positivity, were recorded significantly more often in children between 1 and 6 years of age than in older children, thus serving as further risk predictors in addition to their classification as high-risk patients based on young age (19).

Less than 2% of study patients were POPO4-positive, i.e., presented with severe growth retardation (< −2.5 SDS), in significant contrast with data from the EPIMAD registry which pointed to growth retardation (< = −2 SDS) in 9.5% of patients (25 of 261) (20) and reports on incidences of growth failure as high as 15–40% (21). The variability of these incidences might be explained in part by the use of different reference systems and cut off values.

We found that severe growth retardation at diagnosis had an extremely high PPV for persisting growth failure. Because growth retardation in paediatric CD reflects contributing pathogenetic factors such as malnutrition, anorexia and inflammation (22) and intensified therapy with early use of biologics can lead to increased catch up growth (23–25), our data substantiates even further the necessity for more aggressive treatment at an early stage. POPO-4-positive patients had also very high PPVs for lack of sustained remission >1 year and EIM, relevant negative outcome parameters contributing substantially to long-term morbidity (19, 26). In retrospective studies, an increased risk for surgery was associated with lower weight Z-scores (27). Since Z-score deviations in juvenile patients may have causes other than CD we chose a stricter cut-off for this study.

4.3% of the cohort studied had stricturing or penetrating disease (POPO-6-positive), in contrast to reports from France and Scotland, with 29 and 9% of patients showing B2/B3 behaviour at diagnosis, resp., (18, 28) and even higher rates in the Swiss IBD Cohort Study Group (29). These data reflect disease complications over time rather than at diagnosis. In fact, a retrospective analysis of small bowel imaging within 30 days of diagnosis in more than 200 paediatric CD patients showed the majority of increased surgical risk to occur in the first year after diagnosis, with a peak percentage as high as 17% (30). The patients we observed must thus certainly be classified as high-risk patients: Not only did they present with a severe complication at an early phase, but, once again in accordance with reports of others (17, 22–24), we found their risks for developing abscess, fistula or stenosis as well as surgery to be significantly higher, and significantly, the PPV for lack of sustained remission >1 year was highest of all POPO-groups.

This was also true for patients with perianal disease (POPO-7-positive), thus supporting the notion of perianal disease at diagnosis as a strong predictor of unfavourable outcome (19). The influence of perianal disease on developing abscess, fistula or stenosis has so far yielded conflicting results in the literature, with reports on an increased risk (OR 3.5 [1.98–6.20]) (15), not confirmed by others (31). However, a recent study from the Swiss IBD Cohort Study Group revealed higher rates of intestinal complications, including those in the anal region, particularly in paediatric CD patients. In our cohort we also found very high ORs for developing abscess, fistula or stenosis (OR 7.36 [4.93–11.0]) as well as raised ORs for later surgery (2.56 [1.58–4.15]), thus further substantiating POPO-7-positivity as a valuable clinical predictor of complicated disease.

Limitations of our study include that other therapeutics strategies were not included, that a substantial number of patients had a followup of <2 years and that we did not collect information on ethnicity, mucosal healing or biosamples. Another problem is group size especially for subgroups. Also, in evaluating the role of medication and outcome, very short time scales cannot be analysed in an observational dataset. We hope to address this by a different model in a following study. The one main limitation of this study is that it is observational. One might also argue that our registry is based on patient data from large regional referral centres, which may not allow extrapolation to the real paediatric IBD population in Germany and Austria. We would, however, go so far as to suggest that this is one of the strengths of our analysis: CEDATA is a multicentre registry with only tertiary centres and IBD specialists participating. We therefore trust the registered data of this carefully selected and large study cohort with patients exclusively diagnosed according to the Porto Criteria to be robust enough to address the risk stratifying potential of the POPO criteria as suggested in the respective guidelines for the first time.

The long observational period of almost 15 years, with a median follow-up of >2 years and 10 visits documented consecutively, was a very important criterion for this research project, since it enabled us to investigate treatment over time with a continuous change in prescription of azathioprine and infliximab, the immunomodulatory and anti-TNF-agents mainly used in our countries.

## Conclusion

This large “real-life” data set showed predictors of poor outcome in paediatric CD to be common. While neither presenting symptoms nor initial disease activity scores were suitable candidates for treatment stratification, the predictors showed significantly increased risks of relevant complications and negative outcomes. Patients with persistent disease at 3 months were prone to fail in reaching sustained remission. A patient who did not respond satisfactorily to initial treatment had a >60% risk of missing this important target. Patients who failed to reach sustained remission carried a significantly increased risk for all complications monitored, therefore sufficient disease control is a mandatory target. Patients with extensive disease had a higher risk of developing abscess, fistula, or stenosis.

Since the group with severe growth retardation showed a very highly increased risk of remaining in growth failure to the end of observation, with two thirds of these patients not reaching sustained remission during follow-up, this should also be a good reason for beginning early and aggressive therapy. The same applies to patients with stricturing or penetrating disease behaviour and with perianal disease, who showed significant increases in fistula, abscess and stenosis development and surgery risk. The problem in how far a “therapeutic window of opportunity” in early CD might be clearly identified and made use of by “treat-to-target” has been addressed only very recently. The intention in this research project was to establish biologics for patients with currently known high-risk factors and also predictors of poor outcomes, suggesting a combination of “phenotype at diagnosis” and “comportment follow-up classification” in the year following diagnosis (32).

## Summary

Stratifying therapy for Pediatric Crohn's Disease requires predictors for negative long-term prognosis. The patient registry data presented demonstrate the value and characteristics of these criteria for the first time in a large real-world pediatric data set.

## Data Availability Statement

The datasets presented in this article are not readily available because; Dataset is part of CEDATA GPGE Registry, cannot be uploaded or transfered, aggregated data is part of the article, additional data is available on request. Requests to access the datasets should be directed to studienzentrale@paediat.med.uni-giessen.de.

## Ethics Statement

Approval for all centers involved was obtained. Written informed consent to participate in this study was provided by the participants' legal guardian/next of kin.

## Author Contributions

JL performed the research, wrote the first draft, and contributed in editing. K-PZ supervised the manuscript and gave valuable insights during editing and analysis. KS provided IT guidance and supported analysis. AH edited analysis, further developed the manuscript, and coordinated the process. All authors contributed to the article and approved the submitted version.

## Conflict of Interest

The authors declare that the research was conducted in the absence of any commercial or financial relationships that could be construed as a potential conflict of interest.

## Publisher's Note

All claims expressed in this article are solely those of the authors and do not necessarily represent those of their affiliated organizations, or those of the publisher, the editors and the reviewers. Any product that may be evaluated in this article, or claim that may be made by its manufacturer, is not guaranteed or endorsed by the publisher.

## Abbreviations

CD: Crohn's disease
IBD: inflammatory bowel disease
PCDAI: pediatric crohn's disease activity index
POPO: predictors of poor outcome
CEDATA: registry for pediatric inflammatory bowel disease
GPGE: gesellschaft für pädiatrische gastroenterologie und ernährung
PPV: positive predictive value
SDS: standard deviation score
NPV: negative predictive value
EIM: extraintestinal manifestation.
