# Supplementary material for: Running Behind “POPO”—Impact of Predictors of Poor Outcome for Treatment Stratification in Pediatric Crohn's Disease
Source: Front Med (Lausanne). 2021 Aug 27;8:644003. doi: 10.3389/fmed.2021.644003 (PMC8430211; doi:10.3389/fmed.2021.644003)

**Tables and Figures as eSupplement**

**Table e1: *Therapy of POPO-positive and -negative patients (first 2 years from diagnosis)***

| **Therapy**  **(Any Medication use documented)** | **1^st^ year** | | | **2^nd^ year** | | |
| --- | --- | --- | --- | --- | --- | --- |
|  | **POPO- positive**  **(n=649)** | **POPO -negative**  **(n=395)** | **p-value** | **POPO- positive**  **(n=477)** | **POPO negative**  **(n=301)** | **p-value** |
|  |  |  |  |  |  |  |
| **Corticosteroids*** | 52.2%  (n=339) | 46.6%  (n=184) | 0.08 | 28.7%  (n=137) | 26.6%  (n=80) | 0.52 |
| **Budesonide (oral)** | 20.6%  (n=134) | 18.7%  (n=74) | 0.45 | 14.5%  (n=69) | 11.3%  (n=34) | 0.20 |
| **Mesalamine** | 66.7%  (n=433) | 59.2%  (n=234) | **0.01** | 59.1%  (n=282) | 51.5%  (n=155) | **0.04** |
| **Sulfasalazine** | 10.3%  (n=67) | 10.6%  (n=42) | 0.87 | 10.3%  (n=49) | 11.6%  (n=35) | 0.55 |
| **Exclusive enteral Nutrition** | 23.1%  (n=150) | 20.5%  (n=81) | 0.33 | 6.1%  (n=29) | 7.0%  (n=21) | 0.62 |
| **Infliximab** | 11.1%  (n=72) | 7.3%  (n=29) | **0.047** | 13.0%  (n=62) | 15.0%  (n=45) | 0.44 |
| **Methotrexate** | 4.2%  (n=27) | 6.8%  (n=27) | 0.058 | 8.8%  (n=42) | 11.6%  (n=35) | 0.20 |
| **Azathioprine** | 64.4%  (n=418) | 61.5%  (n=243) | 0.35 | 66.5%  (n=317) | 65.8%  (n=198) | 0.85 |
| **Probiotics** | 6.2%  (n=40) | 7.3%  (n=29) | 0.46 | 6.1%  (n=29) | 4.3%  (n=13) | 0.29 |
| **Antibiotics** | 19.6%  (n=127) | 15.2%  (n=60) | 0.07 | 8.8%  (n=42) | 4.7%  (n=14) | **0.03** |

* Systemic steroids only

**Table e2: Distribution of POPO criteria among 35 POPO-positive patients of 50 patients between 1-6 years of age**

|  | **POPO2** | **POPO3** | **POPO4** | **POPO6** | **POPO7** |
| --- | --- | --- | --- | --- | --- |
| Pat 1-6 years | N=16 | N=24 | N=3 | N=1 | N=2 |

9/35 patients had 2 POPO-criteria and one patient had 3 POPO-criterion

*ChiSquare /Fisher Exact Test

**Table e3: Symptoms at diagnosis**

| **Symptoms** | **POPO-positive (n=709)** | **POPO-negative (n=375)** | **p** |
| --- | --- | --- | --- |
| **Abdominal pain** | 557 (78.6%) | 278 (74.1%) | 0.11 |
| **Diarrhea** | 467 (65.9%) | 255 (68.0%) | 0.50 |
| **Blood in stool** | 246 (34.7%) | 140 (37.3%) | 0.39 |
| **Failure to gain / weight loss** | 428 (60.4%) | 220 (58.7%) | 0.60 |
| **Growth failure** | 84 (11.8%) | 34 (9.1%) | 0.18 |
| **Fever** | 113 (15.9%) | 56 (14.9%) | 0.73 |
| **Anemia** | 186 (26.2%) | 90 (24.0%) | 0.46 |
| **limitations in activities** | 291 (41.0%) | 129 (34.4%) | **0.03** |
| **Loss of appetite** | 179 (25.2%) | 74 (19.7%) | **0.04** |
| **Extraintestinal manifestation** | 198 (27.9%) | 90 (24.0%) | 0.17 |

**Table e4: Types of surgery performed**

| **Type of surgery performed** | **n** |
| --- | --- |
| Adhesiolysis | 7 |
| Small bowel resection | 7 |
| Exploration / Revision | 5 |
| Abscess/fistula resection | 52 |
| Abscess/fistula drainage | 13 |
| Hemicolectomy left | 1 |
| Hemicolectomy right | 6 |
| Ileocoecalresection | 58 |
| Colectomy | 2 |
| Partial colectomy | 4 |
| Rectal resection | 1 |
| Ostomy placement | 7 |
| Ostomy revision | 4 |
| Stricturoplasty | 2 |
| Abdominal and IBD-related Surgery non specified | 43 |

eSupplement Figure 1 Example - Screenshots CEDATA GPGE online tool


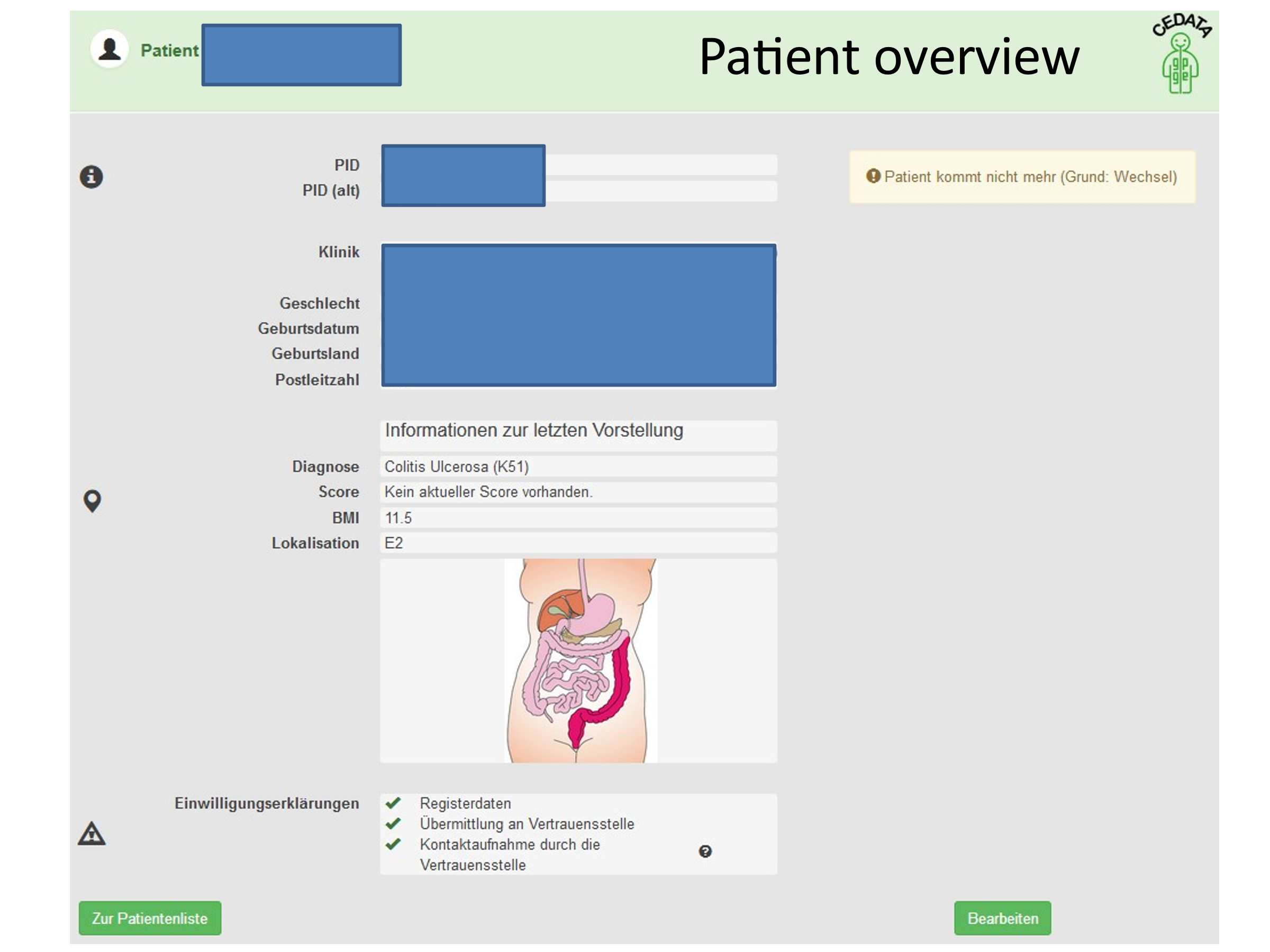


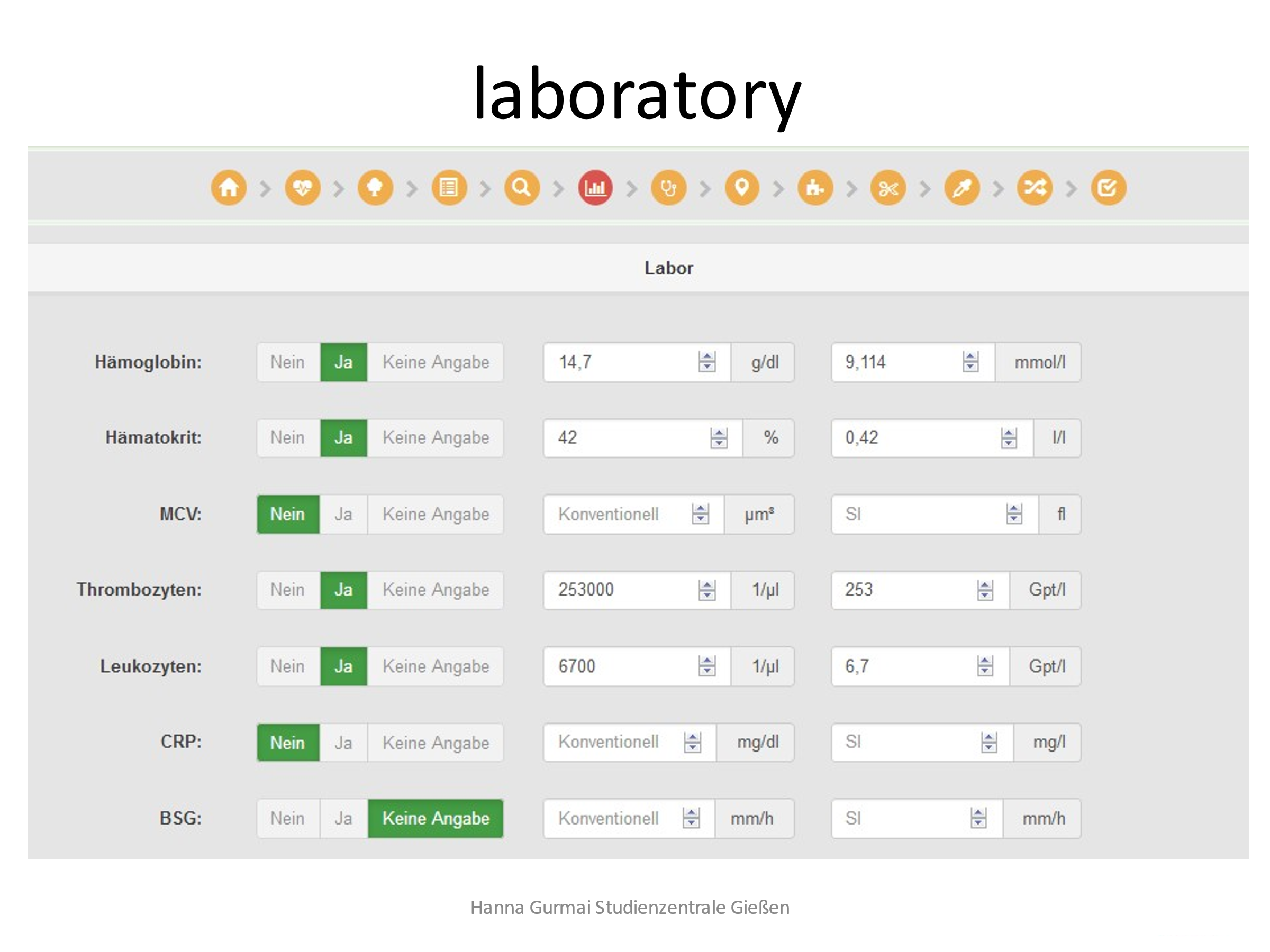

Supplement: Supplementary file 1 [file Data_Sheet_1.docx]
